# Assessment of feeding, ruminating and locomotion behaviors in dairy cows around calving – a retrospective clinical study to early detect spontaneous disease appearance

**DOI:** 10.1371/journal.pone.0264834

**Published:** 2022-03-04

**Authors:** Mahmoud Fadul, Luigi D’Andrea, Maher Alsaaod, Giuliano Borriello, Antonio Di Lori, Dimitri Stucki, Paolo Ciaramella, Adrian Steiner, Jacopo Guccione

**Affiliations:** 1 Vetsuisse-Faculty, Department of Clinical Veterinary Medicine, Clinic for Ruminants, University of Bern, Bern, Switzerland; 2 Department of Veterinary Medicine and Animal Productions, University of Napoli ‘Federico II,’ Napoli, Italy; University of Illinois, UNITED STATES

## Abstract

The study aims to verify the usefulness of new intervals-based algorithms for clinical interpretation of animal behavior in dairy cows around calving period. Thirteen activities associated with feeding-ruminating-locomotion-behaviors of 42 adult Holstein-Friesian cows were continuously monitored for the week (wk) -2, wk -1 and wk +1 relative to calving (overall 30’340 min/animal). Soon after, animals were retrospectively assigned to group-S (at least one spontaneous diseases; n = 24) and group-H (healthy; n = 18). The average activities performed by the groups, recorded by RumiWatch® halter and pedometer, were compared at the different weekly intervals. The average activities on the day of clinical diagnosis (dd0), as well as one (dd-1) and two days before (dd-2) were also assessed. Differences of dd0 vs. dd-1 (ΔD1), dd0 vs. wk -1 (ΔD2), and wk +1 vs. wk -1 (Δweeks) were calculated. Variables showing significant differences between the groups were used for a univariate logistic regression, a receiver operating characteristic analysis, and a multivariate logistic regression model. At wk +1 and dd0, *eating-* and *ruminating-time*, *eating-* and *ruminate-chews* and *ruminating boluses* were significantly lower in group-S as compared to group-H, while *other activity time* was higher. For ΔD2 and Δweeks, the differences of *eating-* and *ruminating-time*, as well as of *eating-*and *ruminate-chews* were significantly lower in group-S as compared to group-H. Concerning the locomotion behaviors, the *lying time* was significantly higher in group-S vs. group-H at wk +1 and dd-2. The *number of strides* was significantly lower in group-S compared to group-H at wk +1. The model including *eating-chews*, *ruminate-chews* and *other activity time* reached the highest accuracy in detecting sick cows in wk +1 (area under the curve: 81%; sensitivity: 73.7%; specificity: 82.4%). Some of the new algorithms for the clinical interpretation of cow behaviour as described in this study may contribute to monitoring animals’ health around calving.

## Introduction

The peripartum period represents a crucial phase for dairy cows’ life cycle because of the significant increase of energy requirements and the severe metabolic adjustments mainly due to the exponential fetal growth, calving and the onset of lactation [1–3]. Throughout this period of physiological and behavioral changes, the natural drop in dry matter intake, as parturition approaches, makes the adaptation phase even more difficult, culminating in a negative energy balance (NEB) status; this is recognized as a risk factor for the development of puerperal diseases [2,4,5]. A successful management of this period, therefore, represents a key factor of profitable farms [6]. As the latter continue to increase in size, and supervision of individual cows gets more difficult, the use of precision dairy farming (PDF) technologies may provide essential support to manage the herd. Their use may increase the overall farm’s efficiency, reduce the time spent for animal management, improve animals’ health and well-being, minimize adverse environmental impact, and sustain high quality of products of animal origin [7–9]. In the past, veterinary involvement in herd health management has been mainly the consequence of farmers’ experience and judgment to identify animals’ behavioral changes [7,10]. Although this skill is still invaluable, it can be easily influenced by human perception and animal’s clinical status (e.g., clinical symptoms not obvious to the human eye or clinical signs only shown at a late stage of disease) [11]. Thus, technologies for monitoring cows’ activities and behavior may have a great impact to support the observations of skilled herdspersons and to allow for early diagnosis improving the success rate of therapeutic measures.

Although the electronic technologies have been primarily developed in attempts to improve estrus detection efficiency, pointing out changes in physical and/or mounting activities (pedometers), the list of devices dedicated to PDF and used for cow status monitoring and management continues to increase, day-to-day [9,12]. Thanks to the rapid development of new technologies and supporting applications, several digital systems have been proposed and validated by different companies and research teams with exciting results [13–15]. Nevertheless, despite widespread availability, the number of information provided by these devices is often limited, and the combined use of multiple technologies becomes less economically feasible for dairy farms requiring a higher level of multiple analytical capabilities at the same time [10].

In this context, RumiWatch® halter (RWh) and the RumiWatch® pedometer (RWp) equipped with three-dimensional (3D)-accelerometers fulfil these criteria, having unique features that–to the best of our knowledge–are not offered by any other accelerometer currently available on the market [16,17]. Indeed, as reported in our previous studies, the combined use of the two devices provides meaningful and accurate information regarding walking, feeding and rumination behaviors in dairy ruminants [18–20]. Sick and healthy cows seem to express different levels of activity [21], including parameters such as number of lying bouts [22], time spent lying down [23], length of strides [24], walking speed [24], chewing activity [25] feeding and rumination time [26,27]. Despite the amount of data available continues to increase, clinical consideration that may arise from them and consequently the systems for the early disease detection still show wide margins for improvement [9]. Promising studies have been focused on changes in feeding behavior as indicators for cows at risk of postpartum disease [28,29]; nevertheless, data analysis investigating the predictive values of pre-partum behaviors on diseases after calving are still incomplete [30,31].

For all these reasons, the current study was initiated. Our hypothesis was that the parameters recorded by the RumiWatch^®^ in dairy cows naturally experiencing the around calving period may represent meaningful tools for reliable animals’ behavior assessment in such a critical period, if associated with the use of new algorithms for data interpretation.

Based on the previous considerations, the goals of the present investigation were: (i) to analyze retrospectively the behaviors of free-stall housed adult dairy cows, before and after calving in states of health and disease, (ii) to compare the RumiWach® output with the findings based on clinical observations, and (iii) to assess the usefulness of newly developed algorithms based on the devices’ data to detect disease.

## Material and methods

### General and ethical animal care

The current investigation was carried out between spring and the beginning of summer 2018 (three months) in a dairy farm located in Caserta district (southern Italy). All clinical and diagnostic procedures performed in this study received institutional approval by the Ethical Animal Care and Use Committee of the University of Naples Federico II [n°2016/0052972] and were performed abiding by the *common good clinical practices* [32] by expert clinicians. Moreover, the farm manager’s consent for scheduled measures and methods used was received before the beginning of the study.

### Farm and management

The study farm was characterized by a free-stall housing system and by an average milk yield/cow of 11127±1558 (kg±SD, 305-days). Forced by the farm routine, animals enrolled were moved through 4 different pens belonging to the same building, during the entire investigation: (i) close-up pen (over a period of three weeks before calving), (ii) calving pen (24h before calving up to the end of the calving event), (iii) post-partum pen (immediately after calving up to 3 days post-partum), (iv) fresh pen (from 4 to 30 days post-partum). All pens were designed to host up to 150 adult dairy cows and to provide at least 10 m^2^ per animal of space (including bedding, feeding and loafing areas). During the entire study period, the stocking density was ≤ 80% of the maximal capacity, the feeding space was ~0.9 m/cow, the linear space for water provision >10cm/cow and the width of passageways if present ≥3m. Cows were ad-libitum fed, twice a day (d), with a total mixed ration specific for the respective reproductive/productive phases (close-up and fresh cows, supplementary file–S1 Table). After calving, all animals were milked 3 times/d, in a side-by-side milking parlour (total of 80 places).

At the investigation time, the farm was already routinely implementing a claw-health monitoring program including: (i) claw trimming 2 times/year; (ii) digital data recording of foot disorders/diseases observed (i.e., location, type and severity); (iii) regular (every three months) locomotion scoring (LS) of the herd according to Sprecher et al.[33]; (iv) in any case of obvious lameness (score ≥3), examination and treatment in the trimming chute was initiated within 48h, for severely lame cows (score = 4 or 5) within 24h. Moreover, stockpersons regularly observed close-up/lactating cows (3 times/d) and cows in the calving pen (every hour, night included) for abnormal behaviors and other signs of disease identification.

### Study design, animal selection, and findings based on clinical observations

The study was designed as observational, longitudinal, and retrospective. The assessment of the clinical usefulness of the output originating from thirteen—continuously recorded—RumiWatch® activities (ITIN + HOCH GmbH, Fütterungstechnik, Liestal, Switzerland) was performed employing 42 healthy, multiparous, Holstein-Friesian, dairy cows [parity = 2.39 ± 1.22; mean ± standard deviation (SD)]. The latter were selected by convenience sampling, therefore, the chosen cows represent all of the available subjects (17.5% of the calving cows) that respected the following eligibility criteria at the selection-time: (i) belonging to the cohort of dry-off cows present in the farm during recruitment (restricted period to minimize seasonal influences on cows’ behaviors) and ranged between 22 to 26 d before the planned calving date (about 80 cows/month); (ii) being classified as healthy and without health problems since the previous transition period time according to the historical data (i.e., free from systemic diseases or from such affecting individual organs); (iii) having a LS ≤2.5 (mean value of 3 independent observers, attributed after observation of a minimum of 10 consecutive strides according to Flower and Weary, [34]); (iv) being without signs of a claw horn lesion or an infectious disease process of the foot, excluded by a functional claw trimming and a complete clinical examination of the locomotor system. Moreover, the good health status was confirmed by (v) a complete veterinary clinical examination (including temperature, respiratory rate, pulse, etc.,) [35] including body condition scoring (BCS), and (vi) a blood sampling (coccygeal venipuncture) for a hemato-biochemical profile analysis [36].

**Fig 1:**
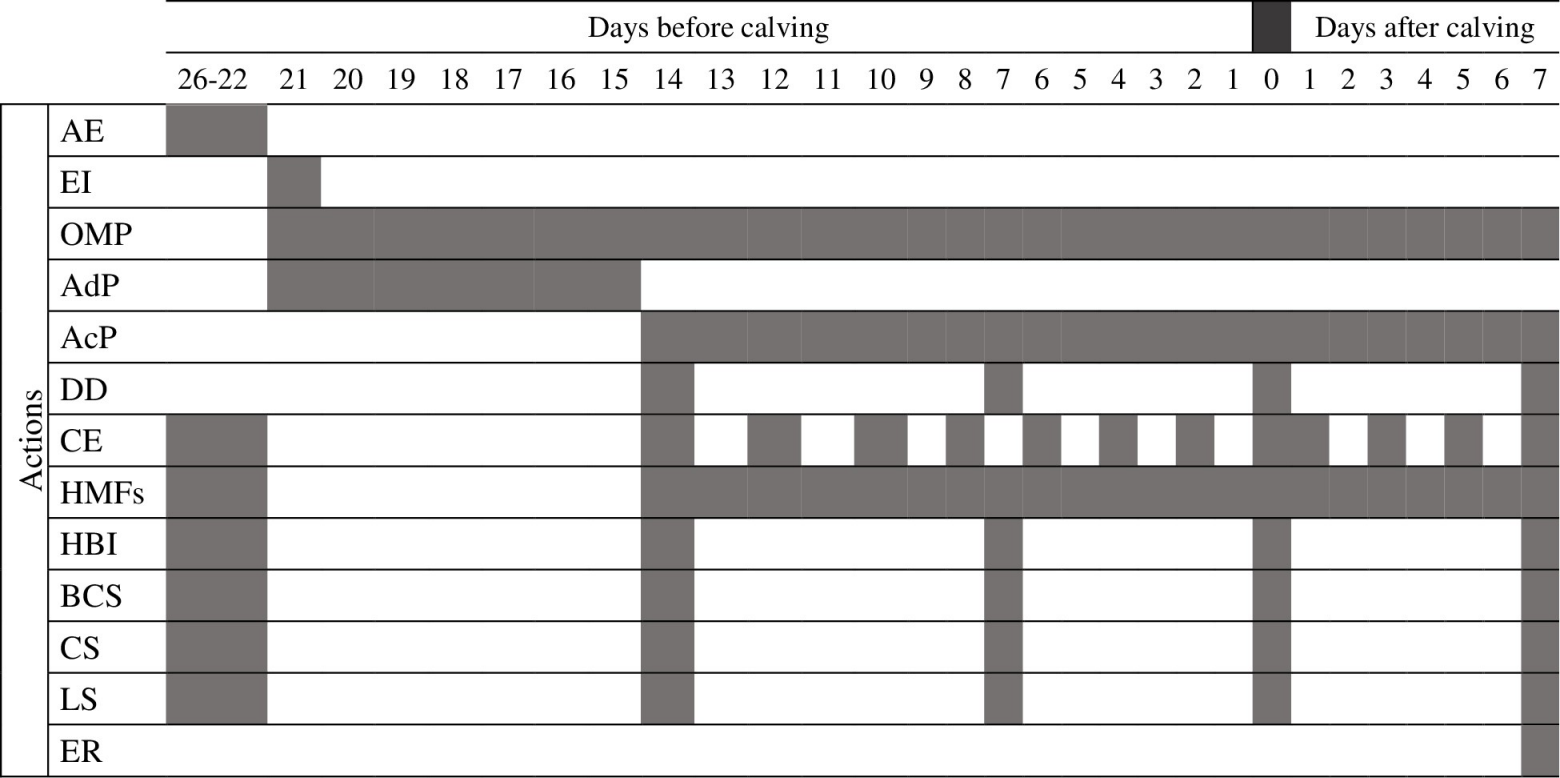
Visual representation and timing of the different actions performed on each cow enrolled. **AE =** animal enrolment; **EI** = equipment installation; **OMP** = overall monitoring phase; **AdP** = adaptation phase; **AcP** = acquisition phase; **DD =** data download; **CE** = clinical examination; **HMFs** = health monitoring by farm-staff three time/d **HBI** = hemato-biochemical investigation; **BCS** = body condition scoring; **CS** = cleanliness scoring; **LS =** locomotion scoring; **ER** = equipment removal.

Actions and timing regarding the findings based on clinical observations are reported in detail in Fig 1. Briefly, the health status was assessed at different time-intervals: (i) daily, by regular health monitoring (observation of cows’ behaviour) routinely performed by the farm-staff (recording abnormalities), and by the investigators during the daily check of the devices; (ii) every other day, by complete clinical examination with particular focus on the overall general status, respiratory, gastro-intestinal, locomotor and genital systems (cows locked at the feeding rack <20min/d, after morning milking); (iii) weekly, by means of BCS [35,37], cleanliness and locomotion scores [34,38], and hemato-biochemical blood analyses (sampling by coccygeal venipuncture) performed directly in farm after collection (BHB and Glucose: FreeStyle Optium, Abbott, Chicago, Illinois, US;- iCa2+ and blood gas analysis: *i*-STAT, Abbott, Chicago, Illinois, US, EG7+ cartridges) and within 1 hour (h) at the University Veterinary Teaching Hospital of the Department of Veterinary Medicine and Animal Productions of Napoli (complete blood cell count—HeCo C–Hematology, Radim Seac, Italy). Exact clinical criteria for diagnosis of the various health disorders observed are given in supplementary file–S3 Table.

Any anomalous cows’ behavior or finding identified by the investigators (during the daily devices-control or the regular clinical examinations), as well as by the farm-staff was further investigated within 1h through in-depth veterinary clinical procedures. Moreover, for the enrolled animals, calving assistance was carried out by the stock-persons and the veterinary surgeons; the presence of delivery problems, potentially modifying animals’ behavior (i.e. unproductive straining for at least 1 h or dystocia) were communicated to the investigators. Finally, data regarding the regular monitoring programs involving the chosen animals (e.g. reproduction, udder health, lameness, nutrition, etc.) and abnormal events were immediately digitally recorded by the farm-staff and made available to the investigators.

### RumiWatch^®^ devices and data handling

Cows included were mounted with both the RWh and RWp d from the 21^th^d before calving up to the 7^th^ after calving (overall 28d) and received a continuous recorders’ monitoring of 40’320 minutes (min). The first 7 d were considered as adaptation phase (10.080 min, from - 21d to - 15d, data not used), while the following 21d (30’240 min, from - 14d to + 7d) were considered as data acquisition phase (data were used for the analysis). Activities performed with the devices were conducted similarly for all cows, and details regarding their timing are reported in detail in Fig 1. Proper functioning of both devices was daily checked by real time monitoring of the cows’ activities transmitted by Wi-Fi connection from the devices attached to the cows to the RumiWatchManager2 software downloaded to a barn-side laptop computer. Both, the RWh and RWp were equipped with an integrated micro SD Memory Card (Swissbit AG, Bronschhofen, Switzerland), where raw data were continuously stored. Weekly, data recorded were transferred from both instruments to the computer by means of an USB cable (supplied by the manufacturer) and stored using RumiWatch^®^ Manager 2 dedicated software (ITIN + HOCH, Liestal, Switzerland). Raw data were converted from ‘.RAW’ filename extension (automatically generated by the software) to ‘.CSV’ filename extension by means of the RumiWatch^®^ Converter V0.7.3.6 (dedicated software, ITIN + HOCH, Liestal, Switzerland). *Eating time*, *ruminating time*, *ruminating boluses*, *eating chews*, *ruminate chews*, *other chews* (i.e., number of chews not attributable to any ruminating, feeding take, or drinking activity), *other activity time* (i.e., time not engaged in eating, ruminating or drinking activities), *lying time*, *standing time*, *walking time*, *lie down*, *stand up*, *number of strides* were the parameters identified by the algorithm and finally reported by the software, as published in previous studies [15,18]), and described in detail in the supplementary file–S2 Table.

### Statistical analyses

Data were expressed as absolute numbers, percentages, or mean ± SD. RumiWatch® output were converted into 1’440-min summaries (24h), and days around calving (4’320 min, d -1, d 0 and d +1) were excluded for the analyses. At the end of the clinical monitoring phase, cows were retrospectively allocated either to group-S (animals affected by ≥1 health disorder) or group-H (no signs of disease observed during the entire study period). The mean values of week-2 (10’080 min, wk. -2: d -14 to d -8), week -1 (8’640 min, wk: -1; d -7 to d -2) and week +1 (8’640 min; wk. +1; d +2 to d +7) relative to calving were calculated. Additionally, the day a disease was first clinically diagnosed (dd0), the day before disease diagnosis (dd-1) and two days before disease diagnosis (dd-2) were defined in group-S. The differences between dd0 and dd-1 (ΔD1), dd0 and week -1 (ΔD2), and between week +1 and week -1 (Δweeks) were also calculated.

Shapiro-Wilk test was performed to check for normality distribution of the variables for each time point and calculated differences separately, and the natural logarithm was calculated for not normally distributed data. To compare between group-H and group-S within wk. -2, -1, +1, dd0, dd-1, dd-2 and for ΔD1, ΔD2 and Δweeks, separately, the equal-variance T-test and Aspin-Welch unequal-variance test for normally distributed variables with equal and unequal variance, respectively were performed. Moreover, for the inter-group comparison, activities performed by cows belonging to group-S at dd0, dd-1, dd-2 were compared with mean daily activities performed by group-H during the corresponding days (for every RW parameter separately). The alpha level of significance was defined as α≤0.05 for all tests; the false discovery rate was considered using the Benjamini-Hochberg procedure, to account for the testing of multiple hypotheses.

Only variables that showed significant differences in the T-test or Aspin-Welch unequal-variance test between group-H and group-S were used for further analysis to determine their usefulness in disease prediction (i.e., detecting cows with a health disorder prior to the traditional clinical diagnosis) or disease detection (i.e., at the very same day as the disease was first clinically diagnosed). Subsequently, univariate logistic regression models were employed to reduce the amount of further potential predictors. To determine the sensitivity (Se) and specificity (Sp) of the model at a given cut-off, a receiver operating characteristic (ROC) analysis was performed. Then, significant variables were combined into multivariate logistic regression models. Only variables moderately or not correlated with each other were combined in the same model (Spearman correlation coefficient > -0.7 and < 0.7). Variables were eliminated from the model by stepwise backward selection. Additionally, the ROC analyses before and after removing a variable were compared to determine how much the variable added to the sensitivity and specificity. Statistical analyses were performed with NCSS® (NCSS12 Statistical Software 2018, LLC. Kaysville, Utah, USA, ncss.com/software/ncss).

## Results

### Animals and findings based on clinical observations

Cows of both groups did not differ in age, parity, milk yield in the preceding lactation and LS around calving; a slight numerical increase in the LS from wk -2 to wk +1, for both groups, was instead observed (Table 1).

**Table 1:** Age, parity, milk yield and lamenss score of healthy cows) and those cows diseased during week +1 relative to calving.

Variables	Group-H^a^ (n = 18)				Group-S^b^ (n = 24)				*P-*value
	Mean	SD	Median	IQR^c^	Mean	SD	Median	IQR	
Age	3.70	0.98	3.39	1.12	3.70	1.11	3.44	1.01	0.99
Parity	2.66	1.32	3.00	1.25	2.25	1.15	2.00	1.75	0.28
Milk yield^d^	11.07	1.61	11.55	1.65	10.58	1.74	10.61	1.80	0.55
LS^e^ week -2	2.14	0.23	2.00	0.50	2.17	0.29	2.00	0.50	0.75
LS^e^ week -1	2.17	0.24	2.00	0.50	2.20	0.25	2.00	0.50	0.59
LS^e^ week +1	2.33	0.34	2.50	0.50	2.29	0.36	2.25	0.50	0.71

^a^Group-H = healthy cows during the entire study period

^b^Group-S = cows diagnosed with at least one health disorder in the first week after calving

^c^IQR = interquartile range

^d^Milk yield = milk yield (kg) in the preceding lactation (*1000)

^e^LS = locomotion score.

In total, clinical procedures included: n = 3’528 behavioral observations; n = 559 clinical examinations, n = 215 hemato-biochemical investigations, n = 215 body condition-, locomotion-, and cleanliness scorings, as well as n = 43 blood gas analyses. A total of n = 47 diseases were diagnosed and included: puerperal metritis (n = 15), subclinical hypocalcemia (n = 7), retained fetal membrane (n = 6), digital dermatitis (n = 4), cecal dilation (n = 3), tracheobronchitis (n = 3), ketosis (n = 2), sole bruise (n = 2), subacute ruminal acidosis (n = 3), interdigital hyperplasia (n = 1), sole ulcer (n = 1). In all cows, the RW devices were well tolerated, and no skin lesions were observed. All health disorders were diagnosed at wk +1, while none at wk -2 and wk -1. Therefore, at the end of the acquisition phase, n = 24 cows were assigned to group-S and n = 18 to group-H. Ten of 24 cows belonging to group-S (41.7%) were categorized as affected by one health disorder and 14 of 24 by more than one health disorders (58.3%). Overall, a least one diagnosis was made at day 1 after calving in 11 of 24 animals (45.8%).

### RumiWacht® data analysis

The overall amount of data used to create new intervals-based algorithms for clinical interpretation of cows behavior originated from 14’206’920 min of continuous recording [(13 activities*42 cows*30’340 min)–(13 activities*42 cows*4’320 min around calving)]. In particular, 8’118’240 and 6’088’680 min were obtained by RWh and RWp, respectively.

Regarding the behavioral intragroup differences between weeks (wks -2, -1 and +1), the overall mean values for feeding, ruminating and locomotion behaviors of both groups S and H, are given in detail in Table 2A and 2B, respectively (descriptive data).

**Table 2a:** Mean values of variables of RumiWatch® halters and pedometers of group-H cows at week -2, -1 and +1.

Variables	Week -2^a^		Week -1^b^		*P-*value	Week -1		Week +1^c^		*P-*value
	Mean	SD	Mean	SD		Mean	SD	Mean	SD	
**Halter**										
Other activity time (min/24 hrs)	525.5	125.2	612.3	206.5	0.15	612.3	206.5	680.5	164.5	0.28
Ruminate time (min/24 hrs)	540.6	84.8	487.8	134.7	0.18	487.8	134.7	439.3	90.9	0.22
Eat time (min/24 hrs)	365.9	78.9	330.7	102.8	0.27	330.7	102.8	311.9	106.6	0.59
Other chews (1000/24 hrs)	1.5	0.7	1.9	0.7	0.07	1.9	0.7	2.0	1.0	0.81
Ruminate chews (1000/24 hrs)	35.0	7.9	30.8	9.3	0.17	30.8	9.3	27.4	6.7	0.21
Eat chews (1000/24 hrs)	24.1	8.1	20.9	9.4	0.29	20.9	9.4	19.8	8.2	0.72
Bolus (bolus/24 hrs)	565.7	82.4	506.4	142.4	0.15	506.4	142.4	426.5	111.9	0.07
**Pedometer**										
Lying time (min/24 hrs)	742.5	124.0	741.0	117.0	0.97	741.0	117.0	582.1	100.2	0.0001
Standing time (min/24 hrs)	659.7	115.8	662.8	112.5	0.93	662.8	112.5	783.4	89.8	0.001
Walking time (min/24 hrs)	38.2	11.8	36.6	9.7	0.67	36.6	9.7	74.9	22.0	< 0.0001
Stand up (unit/24 hrs)	9.4	2.0	9.9	2.4	0.49	9.9	2.4	9.9	2.5	0.99
Lie down (unit/24 hrs)	9.4	1.9	10.0	2.5	0.41	10.0	2.5	9.7	2.8	0.71
Strides (unit/24 hrs)	870.3	273.2	836.1	228.8	0.68	836.1	228.8	2191.4	584.0	< 0.0001

Group-H: Healthy cows during the entire study period

^a^Week-2: From d -14 to d -8 relative to calving date

^b^Week -1: From d -7 to d -2 relative to calving date

^c^Week +1: From d +2 to d +7 relative to calving date.

**Table 2b:** Mean values of variables of RumiWatch® halters and pedometers group-S cows (sick cows) at week -2, -1 and +1.

Variables	Week -2^a^		Week -1^b^		*P-*value	Week -1		Week +1^c^		*P-*value
	Mean	SD	Mean	SD		Mean	SD	Mean	SD	
**Halter**										
Other activity time (min/24 hrs)	531.6	128.4	537.3	113.4	0.88	537.3	113.4	814.5	145.9	<0.0001
Ruminate time (min/24 hrs)	534.2	98.0	524.6	72.8	0.73	524.6	72.8	383.2	105.3	0.00002
Eat time (min/24 hrs)	368.9	69.9	370.6	71.8	0.94	370.6	71.8	234.2	81.3	< 0.0001
Other chews (1000/24 hrs)	1.5	1.0	1.6	0.59	0.91	1.5	0.6	1.9	1.2	0.27
Ruminate chews (1000/24 hrs)	34.1	8.3	33.4	6.8	0.77	33.4	6.8	23.0	7.2	0.00004
Eat chews (1000/24 hrs)	23.9	7.6	23.9	7.7	0.99	23.9	7.7	13.6	6.3	0.00005
Bolus (bolus/24 hrs)	544.7	114.9	531.0	66.1	0.64	531.0	66.1	367.5	110.1	< 0.0001
**Pedometer**										
Lying time (min/24 hrs)	789.5	83.8	774.3	75.3	0.52	774.3	75.3	676.0	90.5	0.0002
Standing time (min/24 hrs)	612.6	79.5	627.5	70.2	0.5	627.5	70.2	700.5	82.1	0.002
Walking time (min/24 hrs)	38.4	8.5	38.6	12.6	0.95	38.6	12.6	63.8	17.4	< 0.0001
Stand up (unit/24 hrs)	9.6	2.2	10.6	3.3	0.28	10.6	3.3	11.4	2.7	0.32
Lie down (unit/24 hrs)	9.7	2.2	10.6	3.3	0.28	10.6	3.3	11.4	2.8	0.35
Strides (unit/24 hrs)	889.8	206.0	887.2	294.8	0.97	887.2	294.8	1803.3	498.1	< 0.0001

Group-S: Cows diagnosed with at least one health disorder in the first week after calving

^a^Week-1: From d -14 to d -8 relative to calving date

^b^Week -1: From d -7 to d -2 relative to calving date

^c^Week +1: From d +2 to d +7 relative to calving date.

Briefly, feeding, ruminating and locomotion behaviors did not differ between weeks -2 and -1 for both groups. In group-H, feeding and ruminating behaviors did not differ between wk -1 and wk +1. The locomotion behaviors *standing time*, *walking time*, and *strides* were significantly higher and *lying time* significantly lower in wk +1 compared to wk -1. In group-S, *ruminating time*, *eating time*, *eating chews*, *ruminate chews*, *and ruminating boluses* were significantly lower and *other activity time* significantly higher in wk +1 compared to wk -1. The locomotion behaviors standing time, walking time, and strides were significantly higher and lying time significantly lower in wk +1 compared to wk -1.

Comparative data between groups within wks regarding feeding, ruminating and locomotion behaviors are given in Table 3.

**Table 3:** Mean values of variables of RumiWatch® halters and pedometers of group-H and group-S at weeks -2, -1 and +1 relative to caving.

Weeks relative to the calving date															
Variables	Week -2^a^					Week -1^b^					Week +1^c^				
	Group-H^c^		Group-S^d^			Group-H		Group-S			Group-H		Group-S		
	Mean	SD	Mean	SD	*P-*value	Mean	SD	Mean	SD	*P-*value	Mean	SD	Mean	SD	*P-*value
**Halter**															
Other activity time (min/24 hrs)	525.5	125.2	531.6	128.4	0.88	612.3	206.5	537.3	113.4	0.16	680.5	164.5	814.5	145.9	0.01
Ruminate time (min/24 hrs)	540.6	84.8	534.2	98.0	0.83	487.8	134.7	524.6	72.8	0.29	439.3	90.9	383.2	105.3	0.09
Eat time (min/24 hrs)	365.9	78.9	368.9	69.9	0.90	330.7	102.8	370.6	71.8	0.17	312.0	106.6	234.3	81.3	0.01
Other chews (1000/24 hrs)	1.5	0.7	1.5	1.0	0.88	1.9	0.7	1.6	0.6	0.08	2.0	1.0	1.9	1.2	0.81
Ruminate chews (1000/24 hrs)	35.0	78.8	34.1	8.3	0.76	30.8	9.3	33.4	6.8	0.33	27.4	6.7	23.0	7.2	0.06
Eat chews (1000/24 hrs)	24.1	8.1	23.9	7.6	0.93	20.9	9.4	23.9	7.7	0.28	19.8	8.2	13.7	6.3	0.01
Bolus (bolus/24 hrs)	565.7	82.4	544.7	114.9	0.54	506.4	142.4	531.0	66.1	0.49	426.5	111.9	367.5	110.1	0.11
**Pedometer**															
Lying time (min/24 hrs)	742.5	124.0	789.5	83.8	0.16	741.0	117.0	774.3	75.31	0.27	582.1	100.2	676.0	90.5	0.003
Standing time (min/24 hrs)	659.7	115.8	612.5	79.5	0.13	662.8	112.5	627.5	70.23	0.22	783.4	89.8	700.5	82.1	0.003
Walking time (min/24 hrs)	38.2	11.8	38.4	8.5	0.94	36.6	9.7	38.6	12.60	0.59	74.9	22.0	63.8	17.4	0.07
Stand up (unit/24 hrs)	9.4	2.0	9.6	2.2	0.72	9.9	2.4	10.6	3.28	0.49	9.9	2.5	11.4	2.7	0.07
Lie down (unit/24 hrs)	9.4	1.9	9.7	2.2	0.68	10.0	2.5	10.6	3.31	0.55	9.7	2.8	11.4	2.8	0.05
Strides (unit/24 hrs)	870.3	273.2	7.1	31.8	0.80	836.1	228.8	887.2	294.9	0.54	2191.4	584.0	1803.3	498.8	0.02

^a^Week -2: From d -14 to d -8 relative to calving date

^b^Week-1: From d -7 to d -2 relative to calving date

^c^Week +1: From d +2 to d +7 relative to calving date

^d^Group-H: Healthy cows during the entire study period

^e^Group-S: Cows diagnosed with at least one health disorder in the first week after calving.

No significant differences were detected between the two groups within wks -2 and -1. Instead, during wk +1, cows of group-S spent significantly less time *eating* and performed fewer *eating chews* compared to group-H. Moreover, sick animals spent significantly more time doing *other activity* compared to the healthy ones. Concerning the locomotion behavior, cows of group-S walked a significantly lower *number of strides* and spent significantly less time *standing* as compared to group-H. Consequently, sick cows spent significantly more time *lying* than healthy ones.

### RumiWatch® vs. clinical observation based activity

Regarding behavior at dd0, dd-1 and dd-2, data are reported in detail in Table 4.

**Table 4:** Mean values of variables of RumiWatch® halters and pedometers of group-H and group-S cows at dd0, dd-1, and dd-2.

Days relative to the first day of disease diagnosis															
Variables	dd-2^a^					dd-1^b^					dd0^c^				
	Group-H^d^		Group-S^e^			Group-H^d^		Group-S^e^			H group		Group-S^e^		
	Mean	SD	Mean	SD	*P-*value	Mean	SD	Mean	SD	*P-*value	Mean	SD	Mean	SD	*P-*value
**Halter**															
Other activity time (min/24 hrs)	697.2	192.5	711.0	136.3	0.88	684.6	186.7	747.9	163.6	0.39	666.7	160.8	840.7	179.8	0.005
Ruminate time (min/24 hrs)	423.3	117.0	429.5	60.6	0.91	454.4	113.6	421.3	58.7	0.41	438.8	104.8	345.0	147.4	0.04
Eat time (min/24 hrs)	309.1	125.5	294.1	148.7	0.82	291.1	131.2	265.1	140.2	0.64	328.2	126.1	250.3	71.8	0.03
Other chews (1000/24 hrs)	2.2	1.3	1.7	1.1	0.38	2.0	1.2	2.0	1.6	0.9	1.8	1.0	2.3	1.4	0.29
Ruminate chews (1000/24 hrs)	26.6	8.7	25.9	3.7	0.87	28.4	8.3	26.1	4.8	0.45	27.4	7.2	20.7	9.4	0.02
Eat chews (1000/24 hrs)	18.9	9.2	19.2	9.5	0.96	17.8	9.4	16.2	8.8	0.67	21.1	9.3	14.1	5.3	0.01
Bolus (bolus/24 hrs)	417.3	135.9	384.8	40.1	0.6	442.7	135.9	405.6	73.0	0.44	425.5	107.4	335.4	146.3	0.04
**Pedometer**															
Lying time (min/24 hrs)	559.6	115.6	681.5	69.0	0.01	603.4	167.1	637.6	136.2	0.56	604.6	112.8	642.5	161.1	0.41
Standing time (min/24 hrs)	798.3	108.4	691.9	55.7	0.01	763.5	152.2	730.2	119.1	0.53	763.8	92.8	721.5	134.6	0.27
Walking time (min/24 hrs)	82.4	24.3	66.9	24.2	0.15	73.4	24.8	72.4	26.7	0.91	72.1	33.6	76.3	35.5	0.71
Stand up (unit/24 hrs)	10.3	3.3	11.1	2.0	0.51	10.5	4.2	10.8	2.0	0.84	9.8	2.8	11.2	3.5	0.16
Lie down (unit/24 hrs)	10.1	3.1	11.2	1.7	0.33	10.1	3.9	10.6	1.7	0.67	10.0	2.9	11.1	3.6	0.30
Strides (unit/24 hrs)	2374.6	732.6	1972.6	682.4	0.2	2161.4	736.0	2081.8	726.3	0.77	2116.8	959.6	2149.1	975.3	0.91

^a^dd-2: Two days before disease diagnosis

^b^dd-1: One day before disease diagnosis

^c^dd0: The day, a disease was first clinically diagnosed

^d^Group-H: Healthy cows during the entire study period

^e^Group-S: Cows diagnosed with at least one health disorder in the first week after calving.

At dd-2, cows of group-S spent more time *lying* and less time *standing* as compared to cows of group-H. At dd-1, no significant differences were detected for any of the behaviors considered. At dd0, cows of group-S spent significantly less time *eating* and *ruminating* and showed significantly lower numbers of *eating chews*, *ruminate chews* and *ruminating boluses* as compared to group-H; other *activity time* was instead significantly increased. Differences between groups concerning locomotion behavior were not found at dd0.

Data regarding ΔD1, ΔD2 and Δweeks are given in Table 5.

**Table 5:** Mean values of variables of RumiWatch® halters and pedometers of group-H and group-S cows at ΔD1, ΔD2, and Δweeks.

Differences between time period relative to the first day of disease diagnosis															
Variables	Δweeks^a^					ΔD2^b^					ΔD1^b^				
	Group-H^c^		Group-S^d^			Group-H		Group-S			Group-H		Group-S		
	Mean	SD	Mean	SD	*P-*value	Mean	SD	Mean	SD	*P-*value	Mean	SD	Mean	SD	*P-*value
**Halter**															
Other activity time (min/24 hrs)	111.4	158.5	279.6	145.4	0.002	97.5	152.3	294.2	206.7	0.003	-41.7	87.8	39.0	225.5	0.23
Ruminate time (min/24 hrs)	-76.7	113.7	-148.7	108.9	0.06	-74.5	130.3	-176.5	151.5	0.04	7.9	65.7	-35.4	147.9	0.34
Eat time (min/24 hrs)	-33.7	91.2	-131.1	82.2	0.001	-20.2	102.2	-114.0	95.0	0.01	36.2	78.9	-2.5	99.0	0.29
Other chews (1000/24 hrs)	0.08	0.96	0.42	1.2	0.34	-0.1	0.91	0.73	1.5	0.06	-1.5	0.5	0.03	1.0	0.56
Ruminate chews (1000/24 hrs)	-5.2	7.6	-11.0	8.0	0.03	-5.0	8.5	-12.8	10.4	0.02	0.4	4.1	-2.6	8.8	0.27
Eat chews (1000/24 hrs)	-2.1	7.7	-10.2	6.5	0.001	-1.0	8.3	-9.9	9.4	0.01	2.9	5.6	-0.4	7.5	0.23
Bolus (bolus/24 hrs)	-109.3	130.9	-168.5	112.7	0.15	-109.4	132.3	-195.6	150.0	0.08	1.2	66.1	-27.2	150.6	0.53
**Pedometer**															
Lying time (min/24 hrs)	-158.9	114.3	-98.3	86.0	0.05	-135.6	139.1	-126.9	151.3	0.85	16.1	132.3	33.6	190.1	0.78
Standing time (min/24 hrs)	120.7	104.4	73.0	76.1	0.09	99.8	128.7	90.0	127.3	0.81	-12.0	131.2	-33.8	164.8	0.71
Walking time (min/24 hrs)	38.3	22.8	25.3	20.1	0.05	35.8	35.6	36.9	36.8	0.92	-4.0	25.9	0.24	33.7	0.71
Stand up (unit/24 hrs)	-0.01	2.0	0.9	3.4	0.33	-0.2	2.9	0.7	4.7	0.50	-0.6	3.2	1.1	3.8	0.23
Lie down (unit/24 hrs)	-0.3	2.5	0.8	3.5	0.23	0.02	2.8	0.6	4.7	0.66	0.02	3.1	1.3	3.2	0.33
Strides (unit/24 hrs)	1355.2	608.4	916.0	552.0	0.02	1289.8	1001.0	1242.1	1003.6	0.88	-104.7	737.7	36.8	980.3	0.67

^a^ΔWeeks: Differences between week +1 and week -1

^b^ΔD2: Differences between the day of dd0 and week -1

^c^ΔD1: Differences between the day of dd0 and dd-1

^d^Group-H: Healthy cows during the entire study period

^e^Group-S: Cows diagnosed with at least one health disorder in the first week after calving.

Significant differences between groups for ΔD1 were not found. For ΔD2 and Δweeks, cows of group-S showed significantly higher differences for *eating time*, *eating chews*, *ruminate chews* and *other activity time* compared to cows of group-H. For *ruminate time*, a significant difference between the two groups was only found for ΔD2. For Δweeks, cows of group-S had a significantly smaller difference in the *number of strides*, *walking time* and *lying time*.

Results of univariable logistic regression models at wk +1, dd-2, dd0 and for ΔD2 and Δweeks are shown in Table 6.

**Table 6:** Results of the univariable logistic regression models and receiver operating characteristics (ROC) analysis of cows being diseased using different RumiWatch® halter and pedometer variables and the cut-off values with highest sensitivity and specificity.

Variable	AUC^b^ (95% CI)	Cut-off	Sensitivity %	Specificity %
**Week +1**				
Other activity time	0.71 (0.50–0.85)	705.6	85.0	47.1
Eat time	0.74 (0.52–0.87)	262.2	64.7	80.0
Eat chews	0.72 (0.49–0.86)	17729.8	70.6	85.0
Lying time	0.76 (0.57–0.87)	652.1	65.2	83.3
Standing time	0.75 (0.56–0.87)	726.0	83.3	69.6
Lie down	0.68 (0.47–0.81)	9.2	87.0	50.0
Strides	0.66 (0.45–0.80)	2260.5	38.9	91.3
**dd-2**					
Lying time	0.84 (0.55–0.95)	662.6	66.7	92.3
Standing time	0.82 (0.54–0.94)	719.7	84.6	66.7
**dd0**				
Other activity time	0.75 (0.53–0.87)	657.7	94.1	52.9
Ruminate time	0.67 (0.44–0.82)	391.2	76.5	58.8
Eat time	0.69 (0.45–0.84)	287.2	64.7	82.4
Ruminate chews	0.71 (0.49–0.85)	23303.0	82.4	52.9
Eat chews	0.73 (0.48–0.87)	17174.0	70.6	82.4
Bolus	0.68 (0.45–0.83)	432.0	58.8	76.5
**ΔD2**				
Other activity time	0.77 (0.57–0.89)	159.4	79.0	89.5
Ruminate time	0.68 (0.46–0.82)	-23.0	47.1	58.8
Eat time	0.79 (0.59–0.90)	-54.2	64.7	79.0
Ruminate chews	0.70 (0.48–0.83)	-8292.6	70.6	63.2
Eat chews	0.79 (0.59–0.90)	-7320.4	76.5	68.4
**Δweeks**					
Other activity time	0.77 (0.57–0.89)	253.1	58.0	82.4
Eat time	0.79 (0.59–0.90)	-54.2	64.7	79.0
Ruminate chews	0.70 (0.48–0.83)	-9315.1	76.5	57.9
Eat chews	0.79 (0.59–0.90)	-8062.4	82.4	63.2
Lying time	0.64 (0.43–0.79)	-142.5	73.9	50.0
Walking time	0.63 (0.43–0.78)	18.3	88.9	43.5
Strides	0.66 (0.45–0.80)	731.8	88.9	47.8

^a^AUC: Area under the receiver operating characteristics curve

^b^ CI = confidence Interval

Although most of the univariable logistic regression models showed a significant association of the feeding, ruminating and locomotion variables with the presence of health disorders in wk +1, they had a sensitivity or specificity of less than 80%. *Eating chews* and *standing time* during wk +1 were the variables with the highest sensitivity and specificity for differentiation between groups. Indeed, the models revealed an area under the ROC-curve (AUC) of 0.72 and 0.75, a sensitivity of 70.6% and 83.3% and specificity of 85% and 69.6%, for the two variables considered. The univariable models for *eating chews*, showed an AUC of 0.73, 0.79 and 0.79 for dd0, ΔD2 and Δweeks, respectively. For the same parameter, a sensitivity of 70.6%, 76.5%, and 82.4% and a specificity of 82.4%, 68.4%, and 63.2%, respectively, was found.

The results of the multivariable logistic regression models are shown in Table 7.

**Table 7:** Combination of the different Rumiwatch® noseband sensor and pedometer variables as predictors of cows being sick in multivariable logistic regression and receiver characteristics analysis on different cut-off values with corresponding sensitivity and specificity.

Variable	AUC^a^ (95% CI^b^)	Cut-off	Sensitivity %	Specificity %
**Week +1**				
Eat Time	0.75 (0.53–0.88)	262.2	64.7	84.2
+ Lying time	0.74 (0.53–0.86)	652.1	57.9	88.2
	0.77 (0.56–0.89)	0.5	79.0	64.7
Eat Time	0.75 (0.53–0.88)	262.2	64.7	84.2
+ Lying time	0.74 (0.53–0.86)	652.1	57.9	88.2
+ Strides	0.62 (0.39–0.77)	2225.5	41.2	89.5
	0.78 (0.57–0.89)	0.5	73.7	70.6
**dd0**				
Eat chews	0.67 (0.44–0.82)	391.2	76.5	58.8
+ Ruminate time	0.73 (0.48–0.87)	17174.0	70.6	82.4
	0.76 (0.54–0.88)	0.4	82.4	64.7
**ΔD2**				
Eat chews	0.79 (0.59–0.90)	-8062.4	82.4	63.2
+ Ruminate chews	0.70 (0.48–0.83)	-9315.1	76.5	57.9
	0.78 (0.56–0.90)	0.6	63.2	88.2
Eat chews	0.79 (0.59–0.90)	-8062.4	82.4	63.2
+ Ruminate chews	0.70 (0.48–0.83)	-9315.1	76.5	57.9
+ Other activity time	0.77 (0.57–0.89)	197.0	68.4	70.6
	0.81 (0.60–0.92)	0.5	73.7	82.4
**Δweeks**				
Other activity time	0.79 (0.59–0.90)	159.4	83.3	58.8
+ Strides	0.62 (0.39–0.78)	731.8	88.2	38.9
		0.80 (0.59–0.91)	0.4	83.3	64.7

^a^AUC: Area under the receiver operating characteristics curve

^b^ CI = confidence Interval.

For ΔD2, the model considering the combination of the variables *eating chews* and *ruminate chews* revealed the highest accuracy in detecting cows with health disorders with an AUC of 0.78, a sensitivity of 63.2% and specificity of 88.2%. Adding the variable *other activity time* from the noseband sensor, the AUC and sensitivity increased up to 0.81 and 73.7%, respectively, while specificity decreased to 82.4%. For Δweeks, the model with the highest accuracy to detect cows with one or more health disorder(s) considered variables from both the noseband sensor and the pedometer; the combination of *other activity time* and *strides* allowed to reach an AUC of 0.80, a sensitivity of 83.3% and specificity of 64.7% for the differentiation between groups S and H.

## Discussion

Estimation under field conditions of the clinical usefulness of the output originating from the novel dairy farming technology can represent a stimulating challenge in cows’ medicine, where the knowledge regarding behavioral changes over time is still incomplete, even more, when associated with spontaneous diseases appearance. Therefore, the goal of the present investigation was to evaluate whether the RumiWatch® output around calving may represent supporting bases for new meaningful clinical considerations if analyzed with new time intervals and compared to findings based on clinical observation.

During the entire study period, one of the main positive aspects was represented by the good tolerance of the cows to RWh and RWp. Indeed, none of the cows showed signs of lacerations or lesions due to their long-term positioning of the instruments (mainly employed for scientific purposes), even longer in place than previously described in the literature [19,39]. Although an apparently low number of animals has been enrolled, a relevant amount of data has been produced during the study both by the findings based on clinical observations and examinations and by the continuous digital monitoring based on thirteen variables/cow of two tools simultaneously used (Supplementary file–S2 Table). Despite the fact that the major part of previous studies concerned sensors-based validations [15,40,41], observations of physiological behaviors [20,42,43] or comparison between PDF technologies outputs and the available animals’ health parameters derived from the farms’ databases [29,44,45], only a few studies focused on a strict comparison between the devices’ output and individual clinical-diagnostic activities [46–48], such as performed in the current investigation (Fig 1 and Supplementary file–S3 Table).

Based on clinical observations, group-S was found to be heterogeneous concerning the diseases, because several common and concomitant diseases of the peripartum period were observed. Although, a retrospective categorization, according to a single pathology was not possible, the analysis of the RumiWatch® output revealed some interesting differences between the two groups. Our findings appeared in accordance with what was described by several authors, assessing significant differences regarding ruminating and feeding behaviors, not obviously dependent on the type and severity of the diseases [19,49–51].

The comparison between intra-group weekly activities, made possible to draft interesting considerations. Although the current study did not show any intra-group differences in feeding, ruminating and locomotion behaviors for the whole population studied in wk -2 and wk -1, changes occurred from wk-1 to wk+1 (Table 2A and 2B). It was already demonstrated that *lying time* and *lying bouts’* duration significantly decrease, while *standing time* increases, in healthy cows from wk -1 to wk +1 [49,52]. Our results, comparing the locomotion activities between the same periods, additionally indicated that *walking time* and *number of strides* significantly increased in both groups (Table 2A and 2B). The resumption of milk production (associated with the rise of nutritional requirements and time spent searching for food), the necessity to walk to the milking parlor 3 times/d, as well as the regrouping of cows in other barns might explain the significant changes for these parameters observed within both groups. The authors realized how such an organized herd management system might paradoxically mitigate the early diagnostic ability of the PDF technologies. Indeed, sharing the same environment, having the same time budget to perform certain behaviors and duties, or suffering the same competition from other cows represented some hindrances that influenced cows’ behaviors regardless of their state of health. For rumination and feeding activities, some intra-group differences were instead detected over time (from wk -1 to wk +1). A significant decrease in feeding (i.e., *eating time*, *eating chews*, *boluses*) and ruminating activities (i.e., *ruminating time*, *ruminate chews*, *other activity time*) was indeed recorded in group-S (Table 2A and 2B), but no difference of eating and ruminating variables was found for group-H. Our results seem to be in line with what was described by other authors: sick cows are likely to change their feeding behavior several days before the clinical diagnosis [3,7,29], even if affected by a mild form of disease [49–51]. Based on the previous statements, changes of feeding and ruminating activities between wk. -1 and wk. +1 seem to be candidates to play a role in disease prediction.

Concerning the inter-group comparisons of the weekly activities considered, significant differences were only found for the parameter *other chews* at wk -1 (Table 3). The latter indicates chews attributable to the activity of tongue and mouth in the frame of allogrooming, self-grooming, or licking of surfaces. Miedema and collaborations [53], reporting a consistent increase of ground-licking activity before calving, proved for the first time the usefulness of this behavior as calving predictor. To the authors’ knowledge, a predictive role for disease appearance was not hypothesized so far; therefore, further studies confirming the usefulness of this interesting parameter should be performed in the future. As expected, some feeding (i.e., *eating time*, *eating chews* and *other activity times*) and locomotion behaviors (i.e., *standing time*, *lying time*, *lie down*, *and number of strides*) appeared significantly different between the two groups at week +1, because of the various diseases occurring in group-S (Table 3). The assumption is also supported by the inter-group differences observed for Δweeks regarding some feeding (i.e., *eating time and chews*, *other activity time)*, ruminating (i.e., *ruminate chews)* and walking behaviors (i.e., *number of strides*, *lying time*) (Table 5). As well known [54], cows affected by distress or disease may show several abnormal behaviors and changes of general appearance (e.g., separation from the group, sluggish reaction or indifference to normal stimuli, postural changes, etc.), altering the daily interaction with the environment and consequently influencing the parameters considered in this study. This finding recently received further confirmation, as several authors demonstrated how clinical (e.g., lameness, retained fetal membranes, metritis, etc.) and subclinical diseases (e.g., subclinical ketosis and hypocalcemia, acidosis, etc.) may negatively influence several feeding [7,55], ruminating [29,51,56] and walking behaviors [19,57,58].

About the differences relative to the day of the disease diagnosis based on clinical examination, at dd0 cows of group-S showed different feeding and ruminating behaviors as compared to group-H (i.e., lower eating and ruminating time, eating chews, ruminate chews and ruminating boluses, as well as higher other activity time) (Table 4). Therefore, according to our findings, the RWh seems to be the more reliable device to identify anomalous behaviors originating from one or more diseases, as compared to RWp. To the authors’ knowledge, also other activity time was never considered as potential disease-predictor so far, except for an association with an non-specific discomfort status pre-calving by Fadul and collaborators [39]. Therefore, our outcomes confirm the usefulness of the parameter as effective expression of discomfort-stress in sick animals and its potential to support the in-field clinical examination. At dd-2, two RW variables already indicated an upcoming disease (both from RWp), while at dd0, six variables were significantly different (5 from RWh and 1 from RWp) (Table 4). Nevertheless, the comparison of dd-1 and ΔD1 between group-H and group-S did not reveal any significant difference (Tables 4 and 5). The reason for this observation may be the consequence of a substantial overlap between the three days relative to calving (-1, 0, and +1, excluded from the study) and those of the clinical diagnoses, reducing the overall number of diagnosis useful for the analysis. However, the decision was also endorsed by the farm routine, which planned to move cows from close-up to calving pen 24h before delivery. Indeed, Schirmann and collaborators [21] proved negative effects on rumination and feeding behaviors in cows regrouped immediately before calving. Therefore, based on the previous statement, the poor diagnostic performance of the parameters dd-1 and ΔD1 may be mainly due to the study design rather than RW’s deficiencies. Extending the period of comparison to the entire week before the day of diagnosis (ΔD2), RW seems instead to be able to predict a pathological status with five of the seven parameters belonging to RWh (Table 5). The results of multivariable logistic regression definitively confirm that sick cows differ in a set of behavioral variables from the healthy ones. The analysis revealed satisfying results for ΔD2 (AUC = 0.81, Se = 73.7% and Sp = 82.4%, including *eat chews*, *ruminate chews* and *other activity time*) and Δweeks (AUC = 0.80, Se = 83.3% and Sp = 64.7%, including *other activity time* and *number of strides*), supporting the usefulness of the combined use of RWh and RWp for early diagnosis of clinical diseases in the first week after calving. As recently stated by Knight [12], although the ideal PDF technology still does not exist, those instruments available may actively support herdspersons to identify cows’ behavioral changes, improving their well-being and the overall farm efficiency if immediate adequate measures are taken. Indeed, for example, in order to distinguish between sound and lame cows using RWp and RWh, Beer and collaborators [19] described a very high level of accuracy to discriminate even slightly from non-lame cows by different variables (AUC = 0.96, Se = 100.0% and Sp = 66.7%, including walking speed_calculation, standing bouts and eating time). The explanation of the difference to the current study might be that group-S cows were much more heterogeneous concerning the diseases involved as compared to the lameness study. Therefore, the presence of several pathologies (sometimes even concurrent) may have influenced differently the data originating from the RW sensors. According to the findings of the current investigation, the ΔD2 algorithm seems to adequately anticipate disease diagnoses based on clinical veterinary examination, and it may represent a useful tool supportive to traditional clinical considerations. However, further studies should be performed to confirm this observation.

If one hand, our data analyses open exciting new considerations regarding the clinical use of these algorithms under field conditions, on the other the study has its limits. The first is the unavoidable necessity to exclude d -1, 0, and +1 relative to calving. As known since a long time [59], the myometrial contractions associated with the fetal movements can significantly increase during the 24 hours before calving. The onset of these changes frequently produces signs of discomfort-mild colic, restlessness (with elevated heart and respiratory rates), as well as a fall of the body temperature, potentially influencing animals’ behavior. These findings have been recently confirmed by studies employing PDF technologies and observing how this period can significantly affect rumination and feeding behaviors in dairy cows [21,39]. The second limit was instead represented by the relatively low number of disease-diagnoses and the concomitant presence of multiple diseases affecting the same animals. The current study was based on the observation of the natural behaviors and occurrence of spontaneous diseases of free stall housed adult cows around calving; therefore type, severity and timing of the disease’s appearance were not predictable. Whilst it is true that the study met the necessities exposed by recent manuscripts that suggest both the assessment of the performance of PDF technologies in real situations and the development of new studies focused on the automatic diagnosis of health issues [60,61], it is nevertheless necessary to consider the present study as one of the first examples based on a retrospective clinical trial. The outputs observed should be confirmed in prospective studies, under varying different feeding and husbandry conditions with a larger number of disease events to further assess the reliability of the new algorithms for the clinical interpretation of cow behaviour under field conditions.

## Conclusions

The current retrospective study offers clinical considerations concerning the usefulness of novel algorithms of digitally recorded data of feeding, ruminating and locomotion behaviors in dairy cows naturally experiencing the around calving period. The study revealed that the combined use of RWh and RWp may represent a supportive instrument for clinical interpretation of cows’ behavior, showing alterations of several feeding, rumination and locomotion behaviors at the very same day as the disease was first clinically diagnosed. Moreover, the multivariable logistic regression model of this study revealed that the parameters *eating chews*, *ruminate chews* and *other activity time* achieved the highest accuracy in detecting cows with a health disorder prior to the traditional clinical diagnosis based on veterinary examinations.

The analyses revealed that some of the new algorithms for the clinical interpretation of cow behaviour used may represent a starting point for prospective studies focused on monitoring animals’ health and well-being. In this regard, further studies should be performed to assess the performance of the described algorithms for the clinical interpretation of cow behaviour under field condition.

## Supporting information

S1 Table Ration formulated for close-up and fresh dairy cows by the farm. (DOCX)

S2 Table Definition of the activities recorded by RumiWatch® noseband sensors and 3D-accelerometers. (DOCX)

S3 Table Criteria for clinical diagnosis of the diseases detected in group-S during the entire study period. (DOCX)

S1 Raw data (XLSX)
