# Supplementary material for: Assessment of feeding, ruminating and locomotion behaviors in dairy cows around calving – a retrospective clinical study to early detect spontaneous disease appearance
Source: PLoS One. 2022 Mar 4;17(3):e0264834. doi: 10.1371/journal.pone.0264834 (PMC8896666; doi:10.1371/journal.pone.0264834)
Supplement: S1 Table — (DOCX) [file pone.0264834.s001.docx]

### Supporting information

**S1 Table.** Ration formulated for close-up and fresh dairy cows by the farm.

| **Close up**  *(from 21 days pre-calving to calving day)* | | | **Fresh Cows**  *(from calving day to 30 days in milk)* | | |
| --- | --- | --- | --- | --- | --- |
| Contents | % D.M. | Weigh DM/head  (kg) | Contents | % D.M. | Weigh DM/head  (kg) |
| *Straw* | 92.00 | 3.68 | *Straw* | 92.00 | 1.01 |
| *Soybean flour* | 88.00 | 1.32 | *Soybean hulls* | 91.00 | 0.91 |
| *Dry-period supplement* | 97.00 | 0.23 | *Cotton seed* | 90.00 | 1.35 |
| *Calcium carbonate* | 99.50 | 0.10 | [*Extruded soybean*](https://www.sciencedirect.com/science/article/pii/S0022030215005044) | 88.00 | 2.46 |
| *Protein supplement* | 90.14 | 0.63 | *Complementary feed* | 90.49 | 2.13 |
| *Water* | 0.01 | 0.00 | *Protein supplement* | 90.14 | 0.72 |
| *Grain silage* | 29.00 | 3.28 | *Maize flour* | 86.00 | 6.97 |
| *Maize silage* | 32.00 | 3.36 | *Rumen-protected fat supplement* | 97.00 | 0.44 |
|  |  |  | *Sodium bicarbonate* | 95.00 | 0.07 |
|  |  |  | *Molasses* | 74.00 | 0.81 |
|  |  |  | *Water* | 0.01 | 0.00 |
|  |  |  | *Cereal mixture* | 34.00 | 1.80 |
|  |  |  | *Maize silage* | 35.00 | 8.22 |

%=percentage, DM=dry matter
