# Supplementary material for: Assessment of feeding, ruminating and locomotion behaviors in dairy cows around calving – a retrospective clinical study to early detect spontaneous disease appearance
Source: PLoS One. 2022 Mar 4;17(3):e0264834. doi: 10.1371/journal.pone.0264834 (PMC8896666; doi:10.1371/journal.pone.0264834)
Supplement: S2 Table — (DOCX) [file pone.0264834.s002.docx]

### Supporting information

**S2 Table.** Variables of RumiWatch® noseband sensors and 3D-accelerometers. All the activities were continuously recorded for 30’340 minutes divided in 1’440-minutes intervals (24h).

| **^Variable^** | **^Definition^** |
| --- | --- |
|  |  |
| **Halter^a^** |  |
| ^Eat time^ | ^Time spent intake, chewing, and swallowing of feed. Eating: >30 jaw movements/min, unsteady frequency^ |
| ^Eating chews^ | ^Overall number of eating jaw movements^ |
| ^Ruminate time^ | ^Time spent chewing and swallowing of ruminating boluses. Rumination: >30 jaw movements/min, minimum 3 min duration, steady frequency^ |
| ^Rumination chews^ | ^Number of ruminating chews per bolus^ |
| ^Rumination boluses^ | ^Number of ruminating boluses regurgitated^ |
| ^Other activity time^ | ^Time not engaged in eating, ruminating, or drinking activities during non-ingestive related activities. Other activity: characterized by <30 jaw movements/min, unsteady frequency.^ |
| ^Other chews Number of chews not attributable to any ruminating, feeding take, or drinking activity during non-ingestive related activities.^ | |
|  |  |
| **Pedometer^b^** |  |
| ^Stand up^ | ^Event at which the pedometer angle changes its position from an angle >58° toward the vertical axis to an angle <58° toward the vertical axis^ |
| ^Lie down^ | ^Event at which the pedometer angle changes its position from an angle 58° toward the vertical axis for a duration of at least 50 s.^ |
| ^Lying time^ | ^Sum of the duration of all lying bouts within a given recording period. Lying bouts: period with the pedometer in a position exceeding an angle of 58° toward the vertical axis lasting >50 s. Interruption of this pedometer position for less than 50 s is identified and calculated as one stand-up and one lying-down event but not as a separate standing bout. The lying bout is rated as not interrupted.^ |
| ^Walking time^ | ^Sum of the duration of all walking bouts within a given recording period. Walking bouts: Period characterized by at least 3 consecutive strides in the same direction (forward or backward). The period between 2 strides must not exceed 4 s. Walking bouts are rated as separate if the time between 2 strides exceeds 10 s.^ |
| ^Standing time^ | ^Sum of the duration of all standing bouts within a given recording period. Standing bouts: periods during which the cow is in an upright position but not walking; temporary change of the pedometer angle exceeding 58° toward the vertical axis for less than 50 s is neither rated as lying-down and standing-up events nor as an additional lying bout.^ |
| ^Stride^ | ^One forward or backward movement of the limb within a walking bout.^ |

^a^Zehner et al. (2017); ^b^Alsaaod et al. (2015)

**References**

Alsaaod, M., J.J. Niederhauser, G. Beer, N. Zehner, G. Schuepbach-Regula, and A. Steiner. 2015. Development and validation of a novel pedometer algorithm to quantify extended characteristics of the locomotor behavior of dairy cows. J Dairy Res. 98:6236–6242.

Zehner, N., Umstätter, C., Niederhauser, J.J. and Schick, M., 2017. System specification and validation of a noseband pressure sensor for measurement of ruminating and eating behavior in stable-fed cows. Computers and Electronics in Agriculture, 136, pp.31-41.
