# Supplementary material for: Assessment of feeding, ruminating and locomotion behaviors in dairy cows around calving – a retrospective clinical study to early detect spontaneous disease appearance
Source: PLoS One. 2022 Mar 4;17(3):e0264834. doi: 10.1371/journal.pone.0264834 (PMC8896666; doi:10.1371/journal.pone.0264834)
Supplement: S3 Table — (DOCX) [file pone.0264834.s003.docx]

### Supporting information

**S3 Table.** Criteria for clinical diagnosis of the diseases detected in group-S during the entire study period.

| **Disease** | **Clinical-diagnostic procedures** |
| --- | --- |
| **Claw and foot disorders-diseases^a,b,c,e^** | - Locomotion score (lame if LS> 2.5) - Functional claw trimming - Clinical examination of the areas involved |
| **Ketosis^a,c,e,g,h,i^** | - General clinical examination (to assess overall health status) - Body condition score (to assess if the body condition was within the targets) - Blood beta-hydroxybutyrate > 1.4 mmol/l in lactating cows (4-5 h after the start of feeding) - Blood glucose < 3.0 mmol/l for transition cows (during the entire study-period, 4-5 h after the start of feeding) - Blood non-esterified fatty acids > 0.4 mmol/l at the end of pregnancy (just before feeding) - Blood non-esterified fatty acids > 0.7 mmol/l for lactating cows in early lactation (just before feeding) |
| **Subacute ruminal acidosis^a,c,e,g^** | - General and specific clinical examinations (to assess overall and gastro-intestinal health status) - Faecal consistency (score below 3) and faecal sieve test (particles > 0.5 cm) - Rumen juice pH on-farm (pH < 5.5 at daily nadir 5–8 h after total mix ration introduction) |
| **Subclinical Hypocalcemia^a,c,e,f,g,^** | - General and specific clinical examinations (to assess overall and gastro-intestinal health status) - Body condition score (to assess if the body condition was within the targets) - Absence of recumbency - Blood total blood iCa^2+^ ≤1.0 mM (during the entire study-period) |
| **Cecum dilation^a,c,e^** | - General and specific clinical examinations (to assess overall and gastro-intestinal health status) - Ultrasonographic examination (to assess appearance, position, dimensions, diameter and nature of the contents of the caecum) - blood gas analysis (to assess the acid-base status) |
| **Abomasum displacement^a,c,e^** | - General and specific clinical examinations (to assess overall and gastro-intestinal health status) - Ultrasound examination (to assess appearance, position, dimensions, diameter and nature of the contents of the abomasum) - Blood gas analysis (to assess the acid-base and electrolyte status) |
| **Tracheobronchitis^a,c,e^** | - General and specific clinical examinations (to assess overall and respiratory system health status) - Blood gas analysis (to assess pulmonary gas exchange and acid-base status status) - Complete blood cell count |
| **Puerperal Metritis^a,c,d,e^** | - General clinical examination and trans-rectal palpation (to assess overall and female genital system status) - Timing of the problem (≤21 days of calving) - Vaginal examination and uterine discharge observation (fetid and red-brown) - Complete blood cell count |
| **Retained foetal^a,c,d,e^ membrane** | - General clinical examination and trans-rectal palpation (to assess overall and female genital system status) - Vaginal and transcervical examination (to assess foetal membranes presence) - Timing of the problem (membranes still present >24 h after parturition) |

Group-S: cows diagnosed with at least one health disorder in the first week after calving; LS=locomotion score; iCa=ionized calcium; ^a^Dirksen (2004); ^b^Egger-Danner et al., (2016); ^c^Fubini and Divers (2008); ^d^Hudson (2012);^e^Jackson and Cockcroft (2002); ^f^Martinez et al., (2014); ^g^Mulligan et al., (2006); ^h^Oetzel (2004), ^i^Whitaker (1997)

**References**

Dirksen, G. 2004. Malattie del reticolo e del rumine nel bovino ruminante. Pages 396-454 In Medicina Interna e Chirurgia del Bovino. Dirksen G., Grunder H.D., Stober M. Ed. Le Point Veterinaire Italie srl, Milano, IT.

Egger-Danner C, Nielsen P, Fiedler A, Müller K, Fjeldaas T, Döpfer D, et al. ICAR Claw Health Atlas. 2015. http://www.icar.org/wp-content/uploads/ 2016/02/ICAR-Claw-Health-Atlas.

Fubini, S. and T. J. Divers. 2008. Non-infectious Diseases of the Gastrointestinal Tract. Pages 130-199 In Rebhun's - Disease of Dairy Cattle. Divers T.J. and Peek S.F. Ed. Saunders Elsevier Westline Industrial Drive St. Louis, Missouri, USA.

Hudson, C., M. Kerby, J. Statham and W. Wapenaar. 2012. Managing Herd Reproduction. Pages 108-110 In Dairy Herd Health. Green M., Bradley A., Breen J., Higgins H., Hudson C., Huxley J., Statham J., Green L., Hayton A. Ed. Martin Green, University of Nottingham, UK.

Jackson, P. and Cockcroft, P. 2002. The General Clinical Examination of Cattle. Pages: 9-11. In Clinical Examination of Farm Animals. 1st ed. John Wiley & Sons, New York, NY.

Mulligan, F.J, O'Grady, L., Rice, D.A., and M.L. Doherty. 2006. A herd health approach to dairy cow nutrition and production diseases of the transition cow. Anim Reprod Sci. 2006 96:331-53.

Martinez N, Sinedino LD, Bisinotto RS, Ribeiro ES, Gomes GC, Lima FS, Greco LF, Risco CA, Galvão KN, Taylor-Rodriguez D, Driver JP, Thatcher WW, Santos JE. 2014. Effect of induced subclinical hypocalcemia on physiological responses and neutrophil function in dairy cows. J Dairy Sci. 97:874-87.

Oetzel, G.R., 2004. Monitoring and testing dairy herds for metabolic disease. Vet. Clin. Food Anim. Pract. 20, 651–674.

Whitaker, D.A., 1997. Interpretation of metabolic profiles in dairy cows. Cattle Pract. 5 (1), 57–60.
